# Supplementary material for: Reconciling Mining with the Conservation of Cave Biodiversity: A Quantitative Baseline to Help Establish Conservation Priorities
Source: PLoS One. 2016 Dec 20;11(12):e0168348. doi: 10.1371/journal.pone.0168348 (PMC5173368; doi:10.1371/journal.pone.0168348)
Supplement: S1 Dataset — (ZIP) [file pone.0168348.s002.zip › Taxa/Serra Norte/SN_2013/N4E/Lista N4E-45.pdf]

CAVIDADE N4E-0045							
**Classe**	**Ordem**	**Fam/Outros**	**Gên/Outros**	**Espécie**	**S-U**	**U-S**	Zon.
Annelida	Oligochaeta			jovem		5	E
Arachnida	Acari	Argasidae	*Ornithodoros*	sp.1	X		E
Arachnida	Acari	Laelapidae		sp.41	X		E
Arachnida	Araneae	Araneidae	*Alpaida*	aff. *negro*		X	E
Arachnida	Araneae	Corinnidae	*Xeropigo*	sp.1	1		E
Arachnida	Araneae	Ctenidae		jovem	1		E
Arachnida	Araneae	Filistatidae		sp.1	X	X	E
Arachnida	Araneae	Linyphiidae	*Meioneta*	sp.1		X	E
Arachnida	Araneae	Mysmenidae	*Microdipoena*	sp.1		X	E
Arachnida	Araneae	Ochyroceratidae	*Ochyrocera*	sp.3	X		E
Arachnida	Araneae	Ochyroceratidae	*Speocera*	sp.1		X	E
Arachnida	Araneae	Pholcidae	*Mesabolivar*	aff. *cambridgei*	X		E
Arachnida	Araneae	Pholcidae	*Mesabolivar*	sp.1	X		E
Arachnida	Araneae	Pholcidae		jovem	X	X	E
Arachnida	Araneae	Salticidae		sp.2	X	X	E
Arachnida	Araneae	Segestriidae	*Ariadna*	sp.1	X		E
Arachnida	Araneae	Tetragnathidae		jovem		X	E
Arachnida	Araneae	Theridiidae	*Theridion*	sp.1		X	E
Arachnida	Araneae	Theridiidae	*Theridion*	sp.2		X	E
Arachnida	Araneae	Theridiosomatidae	*Plato*	sp.1		X	E
Arachnida	Araneae	Uloboridae	*Miagrammopes*	sp.1	X	X	E
Arachnida	Opiliones	Manaosbiidae	*Saramacia*	sp.1	1		E
Arachnida	Opiliones	Stygnidae	*Protimesius*	*laevis*	1		E
Arachnida	Pseudoscorpiones	Chernetidae	*Spelaeochernes*	sp.1		X	E
Arachnida	Pseudoscorpiones	Chthoniidae	*Pseudochthonius*	sp.1	X	X	E
Entognatha	Collembola	Cyphoderidae		sp.1	X		E
Entognatha	Collembola	Entomobryidae		sp.1	X		E
Entognatha	Collembola	Entomobryidae		sp.4	X		E
Entognatha	Collembola	Paronellidae		sp.1		X	E
Entognatha	Collembola	Paronellidae		sp.4		X	E
Entognatha	Collembola	Poduromorpha		jovem		X	E
Entognatha	Diplura	Campodeidae		sp.1		X	E
Insecta	Blattodea	Blattellidae		jovem	1		E
Insecta	Blattodea	Blattidae		sp.5	1		E
Insecta	Blattodea	Polyphagidae		jovem		4	E
Insecta	Coleoptera	Carabidae		sp.18	X	X	E
Insecta	Diptera	Cecidomyiidae	Cecidomyiinae	sp.	X		E
Insecta	Diptera	Dolichopodidae		sp.	X		E
Insecta	Diptera	Psychodidae	Phlebotominae	sp.	X	X	E
Insecta	Diptera	Sciaridae		sp.	X		E
Insecta	Diptera	Tipulidae	Tipulinae	sp.	X		E
Insecta	Heteroptera	Cydnidae		jovem		X	E
Insecta	Heteroptera	Reduviidae	Reduviinae	sp.	3	3	E
Insecta	Homoptera	Cixiidae		sp.3	X	X	E
Insecta	Hymenoptera	Formicidae	*Brachymyrmex*	sp.1	X		E
Insecta	Hymenoptera	Formicidae	*Paratrechina*	sp.1	X		E
Insecta	Hymenoptera	Formicidae	*Crematogaster*	sp.1		X	E
Insecta	Hymenoptera	Formicidae	*Pheidole*	sp.2	X	X	E
Insecta	Hymenoptera	Formicidae	*Solenopsis*	sp.1		X	E

Insecta	Hymenoptera	Vespidae		sp.1	X		E
Insecta	Hymenoptera	Vespidae		sp.3		X	E
Insecta	Isoptera	Termitidae	*Nasutitermes*	sp.	X	X	E
Insecta	Lepidoptera	Galacticidae		sp.		X	E
Insecta	Lepidoptera	Noctuoidea		sp.1	1	12	E
Insecta	Lepidoptera	Tineoidea		sp.1		X	E
Insecta	Neuroptera	Myrmeleontidae		jovem	X	X	E
Insecta	Orthoptera	Phalangopsidae	*Paraclodes*	jovem	1		E
Insecta	Orthoptera	Phalangopsidae	*Phalangopsis*	sp.	4	12	
Insecta	Psocoptera	Epipsocidae	*Epipsocus*	sp.1		X	E
Insecta	Psocoptera	Epipsocidae	*Epipsocus*	sp.2		X	E
Insecta	Psocoptera	Epipsocidae		jovem	X	X	E
Insecta	Psocoptera	Psyllipsocidae	*Psyllipsocus*	sp.1	X		E
Malacostraca	Isopoda	Armadillidae		sp.1		X	E
Malacostraca	Isopoda	Philosciidae		sp.1		X	E
Turbellaria	Tricladida			sp.2		4	E
Mammalia	Chiroptera	Emballonuridae	*Peropteryx*	*kappleri*	2	1	E
Mammalia	Chiroptera	Furipteridae	*Furipterus*	*horrens*	5		E
